# rAAV expressing recombinant antibody for emergency prevention and long-term prophylaxis of COVID-19

**DOI:** 10.3389/fimmu.2023.1129245

**Published:** 2023-03-30

**Authors:** Ilias B. Esmagambetov, Ekaterina I. Ryabova, Artem A. Derkaev, Dmitry V. Shcheblyakov, Inna V. Dolzhikova, Irina A. Favorskaya, Daria M. Grousova, Mikhail A. Dovgiy, Vladimir V. Prokofiev, Andrey I. Gosudarev, Daria V. Byrikhina, Ilia D. Zorkov, Anna A. Iliukhina, Anna V. Kovyrshina, Artem Y. Shelkov, Boris S. Naroditsky, Denis Y. Logunov, Alexander L. Gintsburg

**Affiliations:** Federal State Budget Institution, National Research Center for Epidemiology and Microbiology Named after Honorary Academician N.F. Gamaleya of the Ministry of Health of the Russian Federation, Moscow, Russia

**Keywords:** recombinant adeno-associated viral vector (rAAV), single-domain antibodies, VHH, COVID-19, SARS-CoV-2, passive immunization, prophylaxis

## Abstract

**Introduction:**

Numerous agents for prophylaxis of SARS-CoV-2-induced diseases are currently registered for the clinical use. Formation of the immunity happens within several weeks following vaccine administration which is their key disadvantage. In contrast, drugs based on monoclonal antibodies, enable rapid passive immunization and therefore can be used for emergency pre- and post-exposure prophylaxis of COVID-19. However rapid elimination of antibody-based drugs from the circulation limits their usage for prolonged pre-exposure prophylaxis.

**Methods:**

In current work we developed a recombinant adeno-associated viral vector (rAAV), expressing a SARS-CoV-2 spike receptor-binding domain (RBD)-specific antibody P2C5 fused with a human IgG1 Fc fragment (P2C5-Fc) using methods of molecular biotechnology and bioprocessing.

**Results and discussions:**

A P2C5-Fc antibody expressed by a proposed rAAV (rAAV-P2C5-Fc) was shown to circulate within more than 300 days in blood of transduced mice and protect animals from lethal SARS-CoV-2 virus (B.1.1.1 and Omicron BA.5 variants) lethal dose of 10^5^ TCID50. In addition, rAAV-P2C5-Fc demonstrated 100% protective activity as emergency prevention and long-term prophylaxis, respectively. It was also demonstrated that high titers of neutralizing antibodies to the SARS-CoV-2 virus were detected in the blood serum of animals that received rAAV-P2C5-Fc for more than 10 months from the moment of administration.Our data therefore indicate applicability of an rAAV for passive immunization and induction of a rapid long-term protection against various SARS-CoV-2 variants.

## 1 Introduction

Coronavirus disease (COVID-19) is a severe acute respiratory illness, recorded for the first time in Wuhan, China at the end of 2019 (1). COVID-19 pandemic was declared March 11, 2020 (2). A severe acute respiratory syndrome coronavirus 2 (SARS-CoV-2) (Betacoronavirus: Coronaviridae) is the causative agent of the disease. More than 579 million confirmed COVID-19 cases and 6.4 million deaths, associated with COVID-19 are registered worldwide at the moment (3).

Vaccination, associated with formation of collective immunity represents an important factor, limiting spread of the disease. At least 198 vaccines are being investigated in the preclinical settings and 170 agents are being tested in clinical trials nowadays (4). Most of vaccines however induce the formation of immunity within several weeks following administration (5–8) and therefore are not applicable for emergency prevention of COVID-19. Monoclonal antibodies represent a more appropriate strategy for emergency prevention and therapy of COVID-19 and are successfully used in the clinic (9). Tixagevimab/cilgavimab (Evusheld™), regdanvimab (Regkirona™), sotrovimab (Xevudy™), bamlanivimab/etesevimab and casirivimab/imdevimab (REGEN-COV™) are currently approved for the clinical usage. Prolonged prophylaxis with these drugs however is not optimal due to their limited circulation time in the human body (10). Given aforementioned limitations of existing treatment approaches, development of a drug suitable for pre- and post-exposure as well as prolonged COVID-19 prophylaxis is still relevant and passive immunization with recombinant adenoviral vectors (rAAV) enabling delivery and prolonged expression of specific antibodies within the organism can serve as a valuable therapeutic strategy. There is evidence demonstrating the efficacy of this approach for induction of prolonged protection against Ebola, Marburg and influenza viral diseases as well as botulinum neurotoxin intoxication (11–15). The choice of rAAV was dictated by their ability to transduce a wide spectrum of cellular targets, low immunogenicity, safety and prolonged persistence in the body as episome (16).

In our previous study we had generated a panel of antibodies specific to the SARS-CoV-2 spike receptor-binding domain (RBD) (17). The most perspective antibodies with the highest neutralizing capacity against the most alarming SARS-CoV-2 variants (Alpha, Beta, Gamma and Omicron) had been selected. In current study we developed an rAAV, expressing a previously generated antibody (clone P2C5) fused to the human IgG1-fragment (P2C5-Fc). The obtained rAAV-P2C5-Fc demonstrated prolonged expression and circulation of P2C5-Fc in serum of transduced mice (more than 300 days). Given as emergency prevention and long-term prophylaxis rAAV-P2C5-Fc enabled full protection of ACE-2 transgenic mice from a lethal dose of SARS-CoV-2 virus. In addition, to study the biodistribution of rAAV upon intramuscular administration, we obtained rAAV-Luc expressing the luciferase gene. It was found that injection of rAAV into the hind limb is followed local tissue transduction and expression of the transgene at the administration site within at least 60 days.

## 2 Material and methods

### 2.1 Cell lines and viruses

Vero E6 (ATCC CRL-1586) cells were maintained in Dulbecco’s modified Eagle’s medium (DMEM, HyClone, Cytiva, USA), supplemented with 10% or 2% of heat-inactivated fetal bovine serum (FBS, Capricorn Scientific, Germany), L-glutamine (4 mM) and penicillin/streptomycin solution (100 IU/mL; 100 μg/mL) (PanEco, Moscow, Russia).

The SARS-CoV-2 variants B.1.1.1 or PMVL-1 (GISAID EPI_ISL_421275) and Omicron BA.5 (hCoV-19/Russia/SPE-RII-25357S/2022) initially isolated from a nasopharyngeal swab were obtained from the State Collection of Viruses of the Gamaleya Center in Moscow and used in both challenge and Microneutralization Assay. Isolation and further propagation were performed in Vero E6 cells in DMEM (HyClone Cytiva, Austria) with 2% heat-inactivated FBS (Capricorn Scientific GmbH, Germany): the cells were infected at multiplicity of infection (MOI) = 0.01 and incubated at 37°C in 5% CO2. The culture medium was collected at 72 h and clarified by centrifugation at 9000 g for 10 min at +4°C. The culture medium containing the virus was aliquoted, frozen and stored at −80°C.

### 2.2 Animal housing conditions

Female BALB/c mice (6 weeks old, weighing 18–20 g) were purchased from “Pushchino breeding facility” (Pushchino, Moscow, Russia) accredited by the Association for Assessment and Accreditation of Laboratory Animal Care (AAALAC International) and maintained at the central animal facility of the Gamaleya Research Center for Epidemiology and Microbiology. Mice were kept at constant temperature (22 ± 2°C) and relative humidity (50%) with 12 h of artificial light per day, housed in individual T2-type cages (eight animals per cage), and fed with dried food and water *ad libitum*.

Transgenic hemizygous female and male K18-hACE2 mice (B6.Cg-Tg(K18-ACE2)2Prlmn/J; 4–5 weeks old) congenic on a C57BL/6 genetic background were initially purchased from the Jackson Laboratory (USA) and subsequently bred according to the official breeding considerations and following the General Terms and Conditions policy. Mice were housed in ventilated ISOCage P and N systems (Techniplast, Italy) for immunological and infection (BSL3 conforming) studies, respectively, with free access to autoclaved drinking water and standard chow diet. All of the experimental procedures were conducted in N.F. Gamaleya National Research Center for Epidemiology and Microbiology in compliance with the Guide for the Care and Use of Laboratory Animals (NIH Publication #85–23, revised 1996), and approved by the local animal ethics committee (protocol #25, 22 Apr 2022) and conducted.

### 2.3 Production and characterization of rAAV

Recombinant viral vectors rAAV-P2C5-Fc, rAAV-B11-Fc and rAAV-Luc were obtained as described previously (15, 18). Briefly, the AAV-DJ plasmid system was used to produce rAAV-P2C5-Fc, rAAV-B11-Fc and rAAV-Luc. (Cell Biolabs, США). Single domain P2C5 antibody was obtained as described (17). The nucleotide sequence encoding P2C5-Fc antibody was synthesized at Evrogen Company (Moscow, Russia). The optimized sequence of the firefly luciferase gene Fluc was obtained from the vector pGL4.51[luc2/CMV/Neo] (Promega). Both sequences were cloned into the rAAV–EGFP Control Vector plasmid instead of the EGFP gene at the EcoRI and XbaI restriction sites, thus obtaining the rAAV-P2C5-Fc and rAAV-Luc plasmids, respectively. rAAV-P2C5-Fc and rAAV-Luc were produced by transient transfections of HEK293 cells (obtained from the cell cultures collection of the Gamaleya National Research Center for Epidemiology and Microbiology) and cultured in a BioFlo 320 bioreactor (Eppendorf, Germany) with a BioBLU 5c disposable vessel (Eppendorf, Germany) filled with Fibra-Cel matrix (Eppendorf, Germany). rAAV-P2C5-Fc and rAAV-Luc purification was performed using an AKTA flux S tangential flow filtration system (Cytiva Life Sciences, USA) and a Hollow Fiber Cartridge, 100 kDa (Cytiva Life Sciences, USA) following affinity chromatography using AVB Sepharose resin (Cytiva Life Sciences, USA) according to the manufacturer’s protocol.

The purity of the obtained rAAV-P2C5-Fc and rAAV-Luc was assessed by SDS-PAGE with 4%−20% Mini-PROTEAN TG Precast Protein Gel (Bio-Rad, USA) under reducing conditions. The number of rAAV-P2C5-Fc and rAAV-Luc genomic copies was determined by the AAVpro Titration Kit (for Real Time PCR) Ver.2 according to the manufacturer’s protocol.

An *in vitro* transduction efficiency of the purified rAAV-P2C5-Fc preparation was evaluated using HEK293 cell culture. Cells were seeded in 96-well microtiter plates containing 100 µL of DMEM media (4 mM glutamine, 10% FBS, 3.8 g/L sodium bicarbonate) at a concentration of 0.5*10^6^ cells/ml and incubation continued for 4 hours. The cells were then infected with rAAV-P2C5-Fc, the cultural medium was collected 48 hours after and used for measurement of P2C5-Fc recombinant antibody levels. An ELISA kit developed at the Gamaleya National Research Center for Epidemiology and Microbiology and registered for clinical use in Russia (P3H 2020/10393 2020-05-18) was used for SARS-CoV-2 RBD-specific IgG analysis. Concentration of the antibody was estimated using optical density of the culture medium samples and standards, represented by purified P2C5-Fc solutions with prespecified antibody concentrations.

### 2.4 Investigation of rAAV distribution in mice

A single intramuscular injection of rAAV-Luc at a dose of 10^10^ gc/animal was used to investigate rAAV distribution in mouse. Intraperitoneal injections of D-luciferin at a dose of 2.5 µg were performed on day 7 and 60 after the rAAV-Luc administration. Then after 5 min exposition bioluminescence in mice was visualized in IVIS Lumina II under isoflurane anesthesia.

### 2.5 Evaluation of *in vivo* protective capacity of rAAV-P2C5-Fc

*In vivo* studies were performed using a mice transgenic for human angiotensin-converting enzyme 2 (ACE-2) receptors (K18-hACE2 mice). A single injection of rAAV-P2C5-Fc into the thigh muscles of the hind limb was performed and the mouse were infected intranasally with SARS-COV2 at a dose of 10^5^ TCID50 per animal. Intact mice were used as controls. Each group consisted of 5 animals. Clinical signs of the disease, such as weight loss were monitored twice daily throughout the experiment. Intact mice or mice treated with rAAV-B11-Fc were used as a negative control.

### 2.6 Determination of viral load in the lungs of infected animals

Determination of viral load in the lungs of mice transduced with rAAV-P2C5-Fc and infected with SARS-CoV-2 B.1.1.1 was performed as described previously (19). Primers for the mouse and hamster beta-actin gene were used to control RNA isolation (mice: F: CTATTGGCAACGAGCGGTTC, R: CGGATGTCAACGTCACACTTC, P: ROX-GCTCTTTTCCAGCCTTCCTTCTTG-BHQ2; hamsters: F: ACTGCCGCATCCTCTTCCT, R: TCGTTGCCAATGGTGATGAC, P: FAM-CCTGGAGAAGAGCTATGAGCTGCCTGATG-BHQ1 (20).

### 2.7 Evaluation of the pharmacokinetic profile of P2C5-Fc antibody expressed after *in vivo* rAAV–P2C5-Fc transduction

A number of BALB/c female mice weighing 18–20 g were treated with a single intramuscular injection of rAAV-P2C5-Fc at a dose of 2*10^11^ gc/mouse. The blood samples were collected from the facial vein before rAAV-P2C5-Fc transduction (point 0) and 1, 2, 3, 7, 14, 21, 45, 60, 90, 120, 150, 180, 210, 240, 270, and 300 days after rAAV-P2C5-Fc transduction. Serum was collected and stored at−20°C until further analysis. Serum samples were taken from three animals per time point. The concentration of P2C5-Fc antibody in collected serum samples was measured as described in section “2.3 Production and characterization of rAAV.”

### 2.8 Evaluation of immunogenicity of rAAV-P2C5-Fc and P2C5-Fc antibody, expressed *via* rAAV-P2C5-Fc transduction

Evaluation of immunogenicity of rAAV-P2C5-Fc and P2C5-Fc antibody, expressed *via* rAAV-P2C5-Fc transduction, was performed as described previously (15). Immunogenicity of rAAV-P2C5-Fc vector was evaluated by the detection of anti-rAAV capsid protein antibodies in the serum of rAAV-P2C5-Fc-treated mice. Immunogenicity of P2C5-Fc antibody, expressed *via* rAAV-P2C5-Fc transduction, was evaluated by the detection of anti- P2C5-Fc antibodies in the serum of rAAV-P2C5-Fc-treated mice. Samples obtained in the section “Evaluation of pharmacokinetic of P2C5-Fc antibody expressed *via in vivo* rAAV-P2C5-Fc transduction” were used for analysis.

### 2.9 Microneutralization assay with Live SARS-CoV-2

Serum samples from rAAV-P2C5-Fc-transduced mice were two-fold serially diluted starting from 1/10 in complete Dulbecco`s modified Eagle medium (DMEM) supplemented with 2% heat-inactivated fetal bovine serum (HI-FBS). All of following manipulations were performed as described previously (17). The minimal neutralizing dilutions of serum were defined as the highest serum titers that completely inhibited CPE of the virus in two or three of the three replicable wells. All experiments were performed in a Biosafety Level 3 facility (BSL-3). Samples obtained in the section “Evaluation of the pharmacokinetic profile of P2C5-Fc antibody expressed after *in vivo* rAAV-P2C5-Fc transduction” were used for analysis. In order to immediately obtain the average value of the virus-neutralizing titer for each point, sera samples from different animals from the same time point were mixed with each other in equal proportions.

### 2.10 Statistical analysis

Data were analyzed using EXCEL 2010, Graphpad Prism 9.0, and ELISA Master (AlkorBio, Russia) software. The Mann–Whitney U-test and the Gehan–Wilcoxon test with a significance level of 0.05 were used to assess intergroup differences in antibody titers and animal survival. Median survival was determined using Kaplan–Meier analysis. The p-value was determined using the t-test and log-rank test.

## 3 Results

### 3.1 Production and characterization of rAAV expressing P2C5-Fc antibody

**Figure 1:**
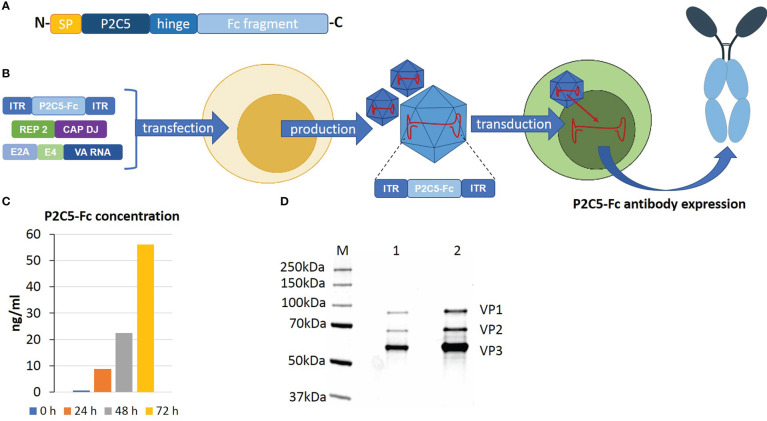
An overview of rAAV-P2C5-Fc preparation and analysis. **(A)** Structure of recombinant P2C5-Fc antibody: SP – amino acid sequence of signal peptide of IgG1 heavy chain; P2C5 – amino acid sequence of P2C5 single domain antibody; hinge – amino acid sequence of hinge region of IgG1; Fc fragment amino acid sequence Fc fragment of IgG1. **(B)** Schematics of rAAV-P2C5-Fc production and mechanism of action. **(C)** Evaluation of rAAV-P2C5-Fc transducing activity using HEK293 cells: bars indicate concentration of P2C5-Fc antibody in cultural media, cultivation time is shown by color. **(D)** SDS-PAGE of an rAAV-P2C5-Fc purity and authenticity analysis: M – protein molecular weight marker; 1, 2 fractions of purified rAAV-P2C5-Fc; VP1, VP2, VP3 rAAV capsid proteins.

P2C5 single-domain antibody specific to RBD of the viral S protein and demonstrating virus neutralization potency was developed as described earlier (17). On the next step an N terminus of the amino acid sequence of the antibody was modified with a signal peptide of a heavy chain of the human IgG1 and a C terminus was modified with an Fc fragment of human IgG1. A P2C5-Fc coding nucleotide sequence was synthesized by Evrogene company and further cloned into pAAV-DJ-vector. rAAV-P2C5-Fc was obtained as described earlier (15, 18). Transducing activity of obtained rAAV-P2C5-Fc as well as expression and specific activity of P2C5-Fc antibody were analyzed using ELISA. An adherent HEK293 cell culture was transduced with 10^2^ gc/cell of rAAV-P2C5-Fc. Level of RBD specific antibodies in cultural medium was analyzed 0, 24, 48 and 72 hours after the transduction using an ‘ELISA SARS Gamaleya’ kit. A range of purified P2C5-Fc concentrations was used as a standard. As a result, an average level of P2C5-Fc antibody in a culture medium of transduced cells reached 8 ng/mL and further increased to 22 and 56 ng/ml 24, 48 and 72 hours after the procedure, respectively. Key steps of rAAV-P2C5-Fc preparation as well as analysis of its purity, authenticity and transducing capacity are shown in (**Figures 1A–D**).

Summarizing all mentioned above, we can say that the generated rAAV expressing P2C5-Fc antibody specific to RBD of the viral S protein, demonstrated high transducing capacity of the vector and showed functional activity of the expressed antibody.

### 3.2 Selection of an optimal rAAV-P2C5-Fc protective dose

**Figure 2:**
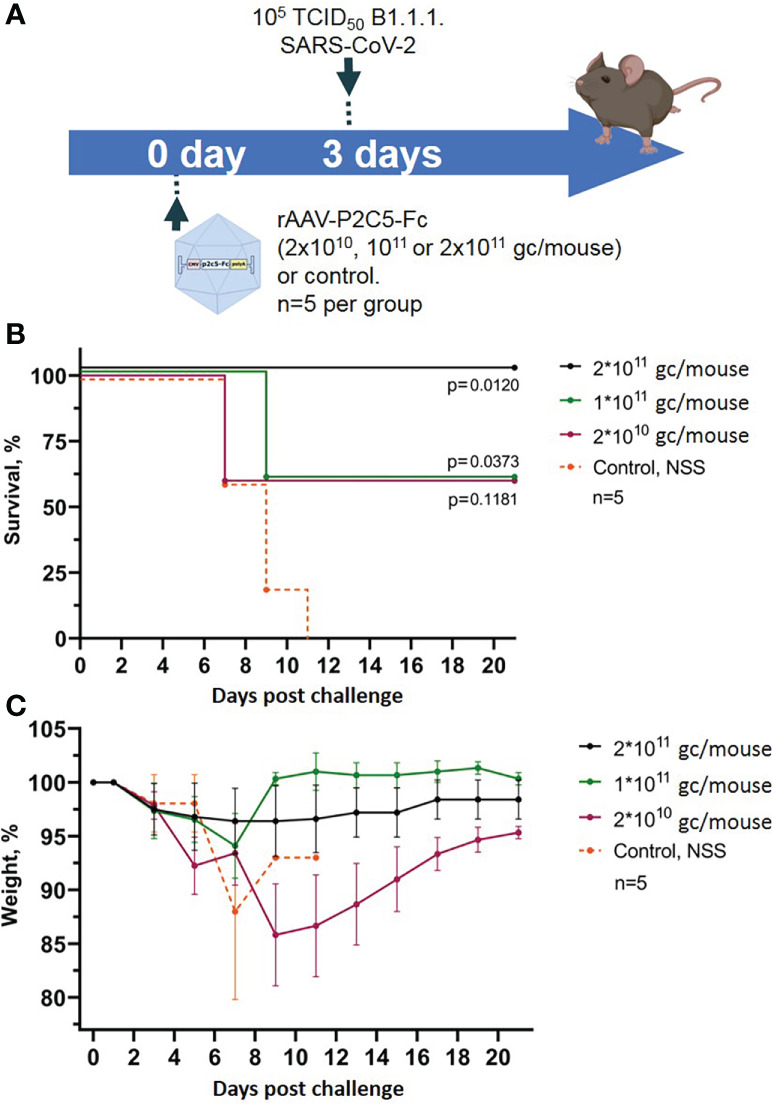
Evaluation of rAAV-P2C5-Fc optimal dose. **(A)** Scheme of the experiment: mice were treated with different doses of rAAV-P2C5-Fc and challenged with 10^5^ TCID50 SARS-CoV-2 B.1.1.1 variant on day 3 after the transduction. **(B)** Animal survival. **(C)** Body weight loss. Lines – mean values, error bars – SD, numbers indicate statistical significance evaluated compared with the vehicle group using a logrank test. Data from different rAAV-P2C5-Fc treatment groups are shown by color. Control, NSS – mice received normal saline solution. n – number of animals per group.

Evaluation of rAAV-P2C5-Fc protective activity was performed using a lethal ACE-2 humanized murine infection model. At the first step selection of an optimal protective rAAV-P2C5-Fc dose was done *via* infection of the experimental animals with a lethal SARS-CoV-2 virus B.1.1.1 variant at a dose of 10^5^ TCID50. Based on the results of the previous studies evaluating protective activity of an rAAV-B11-Fc agent, expressing a recombinant antibody specific to botulotoxin type A (15), as well as observed *in vitro* activity of P2C5-Fc antibody (minimal *in vitro* neutralizing concentration of P2C5-Fc against SARS-CoV-2 virus B.1.1.1 is about 6 ng/ml) rAAV-P2C5-Fc doses of 2*10^10^, 10^11^ and 2*10^11^ gc/mouse were selected. Three days after the rAAV-P2C5-Fc injection animals were challenged with the native SARS-CoV-2 virus. Intact mice (received normal saline solution) were used as a negative control. The scheme of the experiment is shown in **Figure 2A**. As a result, animal survival was 60%, 60% and 100% in groups treated with 2*10^10^, 10^11^ and 2*10^11^ gc/mouse doses, respectively. A significant body weight decrease of more than 10% was observed in animals, treated with 2*10^10^ gc/mouse dose whereas no symptoms of the disease were observed in the group received 2*10^11^ gc/mouse dose. Animal death in the control group was observed 7-11 days after the challenge. Experimental results are shown in (**Figures 2B, C**). Based on the aforementioned observations an rAAV-P2C5-Fc dose of 2*10^11^ gc/mouse or 10^13^gc/kg was considered to be the optimal one and used for further studies.

### 3.3 Evaluation of efficacy of early and emergency pre- and post-exposure prophylaxis with rAAV-P2C5-Fc against SARS-CoV-2 induced disease

**Figure 3:**
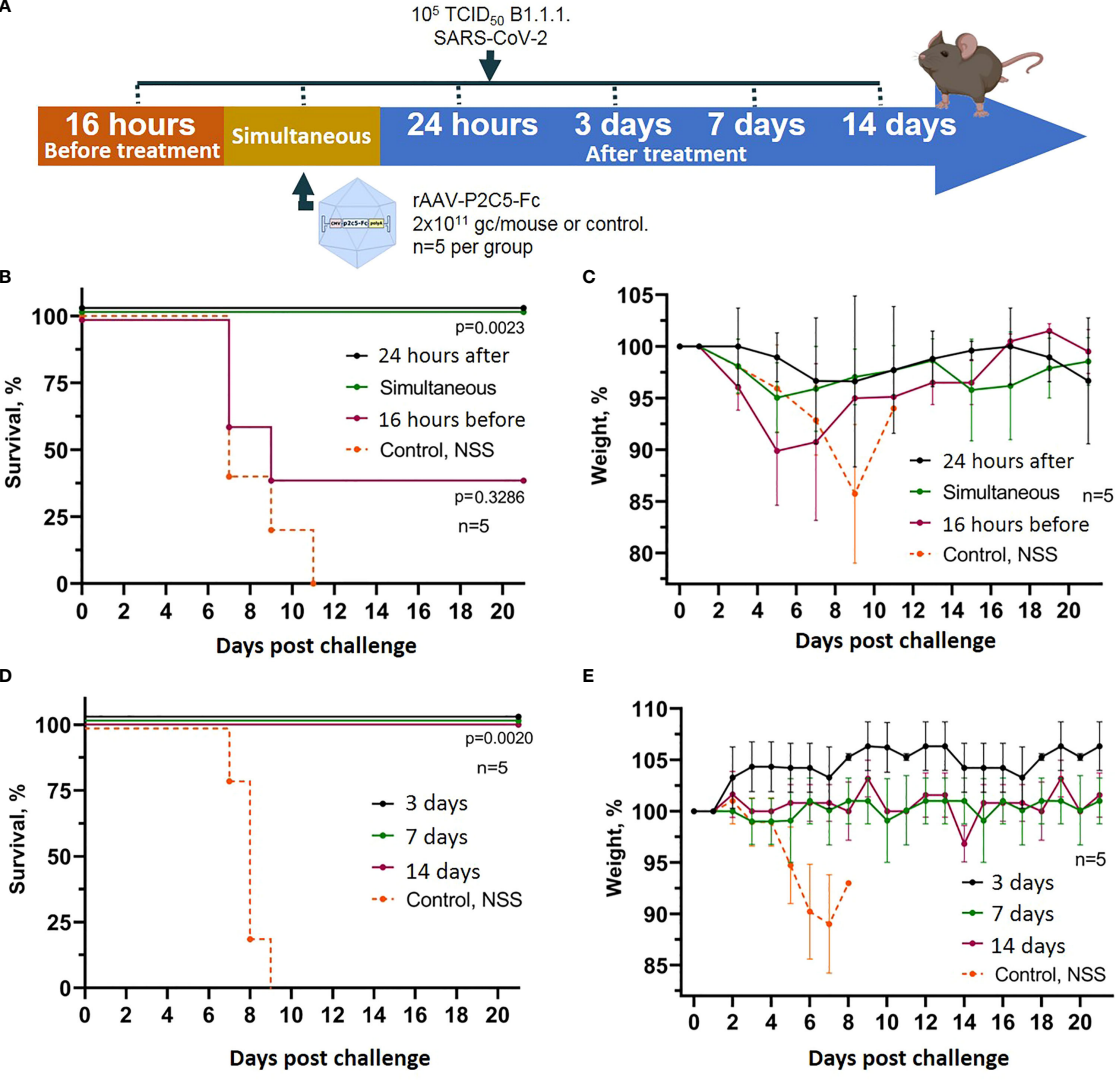
Evaluation of rAAV-P2C5-Fc efficacy for early and emergency prophylaxis and prevention of SARS-CoV-2 infection. **(A)** Scheme of the experiment: briefly, mice were challenged with 10^5^ TCID50 SARS-CoV-2 B.1.1.1 and treated with 2*10^11^ gc/mouse of rAAV-P2C5-Fc at different time intervals. **(B, C)** Survival and body weight loss in emergency prophylaxis animal groups (challenge at 16 hours before, simultaneous with and 24 hours after rAAV-P2C5-Fc transduction). **(D, E)** Survival and body weight loss in early prophylaxis animal groups (transduction at 3,7 and 14 before the challenge). Lines – mean values, error bars – SD, numbers indicate statistical significance evaluated compared with the vehicle group using a logrank test. Data from different rAAV-P2C5-Fc treatment groups are shown by color. Control, NSS – mice received normal saline solution. n – number of animals per group.

To assess the applicability of rAAV-P2C5-Fc for early and emergency pre- and post-exposure prophylaxis of SARS-CoV2 induced disease a humanized ACE-2 mouse model was used analogously to the previous experiment. The animals were transduced with rAAV-P2C5-Fc dose of 2*10^11^ gc/mouce and challenged with 10^5^ TCID50 of SARS-CoV-2 at different time points before or after the treatment. A 16-hour delay between the challenge and rAAV-P2C5-Fc administration was considered as a post-exposure prophylaxis scenario. Pre-exposure emergency prophylaxis regimen implied simultaneous rAAV-P2C5-Fc administration and animal infection; in early pre-exposure prophylaxis groups rAAV-P2C5-Fc was given 24 hours, 3,7 or 14 days before the challenge. Animals that did not receive the rAAV-P2C5-Fc treatment and administrated with normal saline solution were used as a control. The scheme of the experiment is shown in **Figure 3A**. Experimental results indicate a 40% survival and more than 10% body weight decrease in post-exposure prophylaxis group whereas a 100% survival and no weight loss were observed in other groups, treated with rAAV-P2C5-Fc (**Figures 3B–E**). As in the previous study all animals from the control group died within 11 days after the SARS-CoV-2 infection.

**Figure 4:**
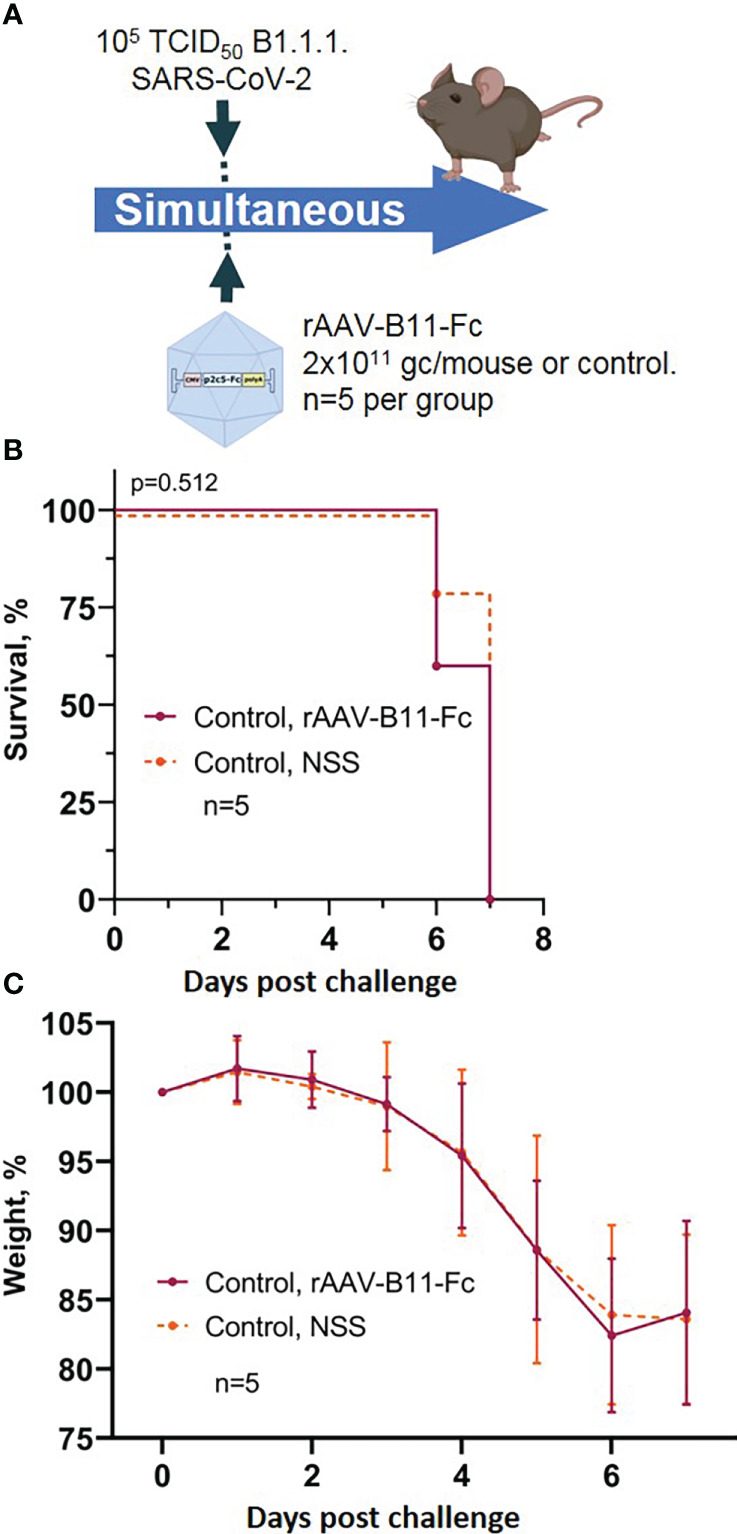
Evaluation of non-specific protective activity of rAAV against SARS-CoV-2 infection. **(A)** Scheme of the experiment: briefly, mice were infected with 10^5^ TCID50 SARS-CoV-2 B.1.1.1 and simultaneous treated with 2*10^11^ gc/mouse of rAAV-B11-Fc. **(B)** Animal survival. **(C)** Body weight loss. Lines – mean values, error bars – SD, numbers indicate statistical significance evaluated compared with the vehicle group using a logrank test. Data from different treatment groups are shown by color. Control, NSS – mice received normal saline solution. n – number of animals per group.

To control the possibility of induction of non-specific protection mediated by rAAV, a group of mice was transduced rAAV-B11-Fc obtained in a previous study (15) and simultaneous infected with 10^5^ TCID50 of SARS-CoV-2. The scheme of the experiment is shown in **Figure 4A**. Animals treated with rAAV-B11-Fc lost weight and fell at approximately the same time as intact mice (**Figures 4B, C**). Thus, the absence of non-specific protection of animals following rAAV administration was demonstrated.

Generated experimental data therefore demonstrate applicability of rAAV-P2C5-Fc for early and emergent pre-exposure prophylaxis of the SARS-CoV-2 induced infection; post-exposure prophylaxis with rAAV-P2C5-Fc showed limited efficacy of 40%.

### 3.4 Evaluation of duration of SARS-CoV-2 induced infection protection with rAAV-P2C5-Fc

**Figure 5:**
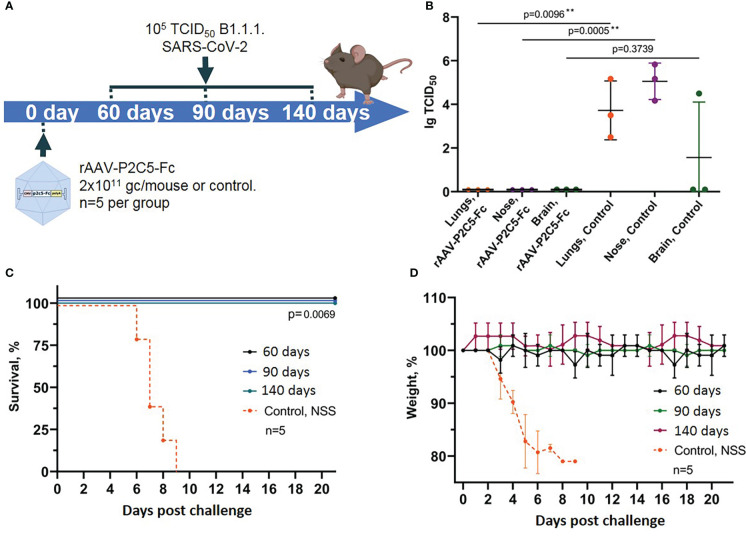
Evaluation of long-term protective activity of rAAV-P2C5-Fc against SARS-CoV-2. **(A)** Scheme of the experiment: mice were treated with rAAV-P2C5-Fc at a dose of 2*10^11^ gc/animal on days 60, 90, 140 before the challenge with SARS-CoV-2 B.1.1.1 variant at a dose of 10^5^ TCID50. **(B)** Viral load in the organs of animals infected with SARS-CoV-2 on day 140 after the treatment. **(C)** Animal survival. **(D)** Body weight loss. Lines – mean values, error bars – SD, numbers indicate statistical significance evaluated compared with the vehicle group using a logrank test (survival) or Mann–Whitney U test (viral load). Data from different treatment groups are shown by color. Control, NSS – mice received normal saline solution. n – number of animals per group. ** means statistically significant.

Duration of protective rAAV-P2C5-Fc action was evaluated using humanized ACE-2 mice. Animals were transduced with a drug dose of 2*10^11^ gc/mouse and challenged with a SARS-CoV-2 infective dose of 10^5^ TCID50 on 60, 90 and 140 days after the transduction. Selection of the time points was done based on our previous studies, where duration of the protective action of a similar preparation against botulotoxin A was shown to be more than 120 days (15). Control mice did not receive an rAAV-P2C5-Fc injection. Scheme of the experiment is shown in **Figure 5A**.

In the current study we evaluated not only animal survival and body weight decrease but also examined sterilizing immunity induced by rAAV-P2C5-Fc. To do so persistence of live virus in lungs, upper respiratory tract and brain of challenged animals was evaluated *via* an *in vitro* titration (**Figure 5B**).

Experimental observations demonstrated full animal protection against SARS-CoV-2 induced infection within at least 4.5 months after the transduction (**Figures 5C, D**). It is very important to note that no signs of the disease as well as no live virus was detected in tissues and organs of the transduced mice, challenged with SARS-CoV-2 at the day 140 after transduction. At the same time high virus concentration was detected in lungs, brain and upper respiratory tract epithelium of the control animals, infected with SARS-CoV-2 virus (**Figure 5B**).

Generated evidence indicated that rAAV-P2C5-Fc induces an immediate animal protection which lasts for at least 4.5 months following the drug injection. In addition, in our study we demonstrated formation of sterilizing immunity preventing virus replication within the body in response to rAAV-P2C5-Fc administration.

### 3.5 Evaluation of protective activity of rAAV-P2C5-Fc against SARS-CoV-2 variant Omicron BA.5

**Figure 6:**
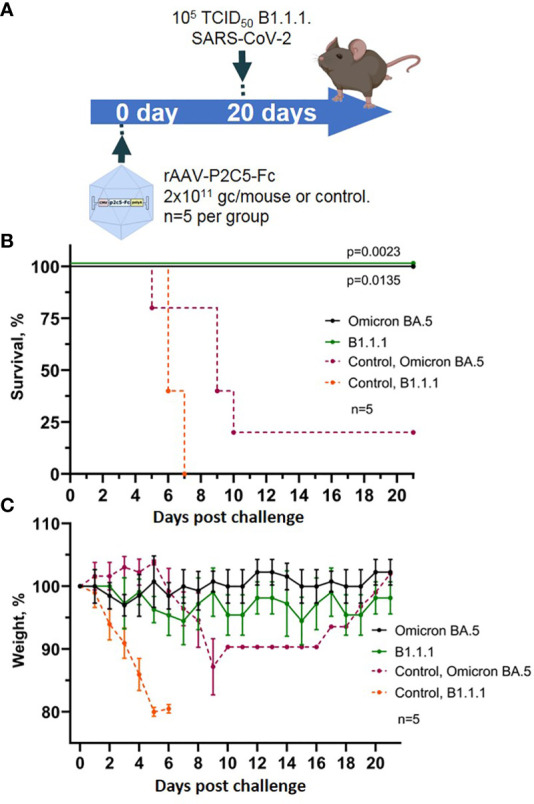
Evaluation of rAAV-P2C5-Fc protective capacities against SARS-CoV-2 variants Omicron BA.5. **(A)** Scheme of the experiment: mice were challenged with 10^5^ TCID50 SARS-CoV-2 variants Omicron BA.5 and B.1.1.1 on day 14 after administration of rAAV-P2C5-Fc at a dose of 2*10^11^ gc/animal. **(B)** Animal survival. **(C)** Body weight loss. Lines – mean values, error bars – SD, numbers indicate statistical significance evaluated compared with the vehicle group using a logrank test. Data from different treatment groups are shown by color. Control, NSS – mice received normal saline solution. n – number of animals per group.

To evaluate effectiveness of our agent against various SARS-CoV-2 variants a humanized ACE-2 mouse model was used. Animals received rAAV-P2C5-Fc at a dose of 2*10^11^ gc/mouse and were challenged with SARS-CoV-2 variant Omicron BA.5 at a dose of 10^5^ TCID50. Control animals, challenged with the virus variants Omicron BA.5 and B.1.1.1 did not receive the treatment. Scheme of the experiment is shown in **Figure 6A**.

As a result, 100% survival was shown in the group of animals challenged with variants Omicron BA.5 and B.1.1.1. and treated with rAAV-P2C5-Fc. In control group of animals, infected with SARS-CoV-2 variant Omicron BA.5, 50% survival and a significant weight loss was observed, whereas all control animals, infected with B.1.1.1 died within 8 days following the challenge. Experimental results are shown in **Figures 6B, C**.

Accumulated data confirms protective activity of rAAV-P2C5-Fc against various SARS-CoV-2 variants including Omicron BA.5.

### 3.6 Evaluation of rAAV biodistribution within the body of transduced mice

**Figure 7:**
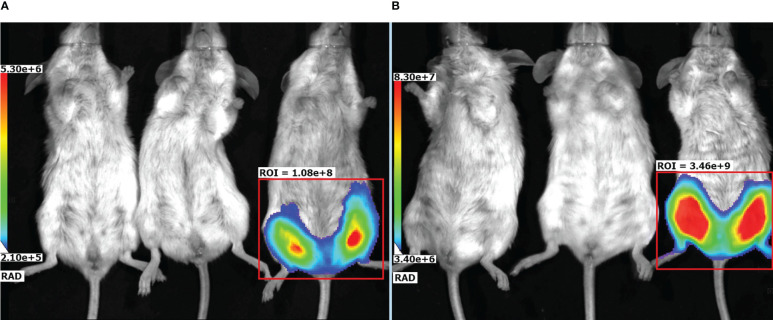
Representative bioluminescent pseudocolored images of luciferase activity in live mice, measured on **(A)** day 7 and **(B)** day 60 after an intramuscular injection of PBS (placebo), rAAV-P2C5-Fc or rAAV-Luc at a dose of 10^10^ gc/mouse into the hind limb. Mice received an i.p. injection of luciferin and was anaesthetized prior to imaging. The intensity of bioluminescence (i.e., luciferase activity) is shown by color.

To evaluate localization of the target gene expression following an rAAV intramuscular injection an rAAV expressing firefly luciferase (rAAV-Luc) was used. BALB/c mice received a single intramuscular injection of rAAV-Luc at a dose of 10^10^ gc/animal into thigh muscles of the hind limb. Intact mice and mice received rAAV-P2C5-Fc, were used as a control group. A luciferase activity was detected solely at the site of rAAV-Luc injection on the day 7 and 60 after the transduction. No luciferase activity was observed in control animals. Experimental results, shown in **Figures 7A, B**, indicate that intramuscular administration of rAAV provides prolonged local expression of the transgene, lasting more than 60 days. In addition, the expression level of luciferase on day 60 was at least 10 fold higher than on day 7.

### 3.7 Evaluation of virus neutralization activity of blood serum obtained from animals, transduced with rAAV-P2C5-Fc

Final stage of rAAV-P2C5-Fc protective activity investigation was dedicated to evaluation of virus neutralization activity of mouse blood serum against SARS-CoV-2 variant B.1.1.1. Serum samples were collected at 0, 1, 2, 3, 7, 14, 21, 45, 60, 90, 120, 150, 180, 210, 240, 270 and 300 days after the transduction. Blood serum of the untreated intact mice was used as a negative control.

**Figure 8:**
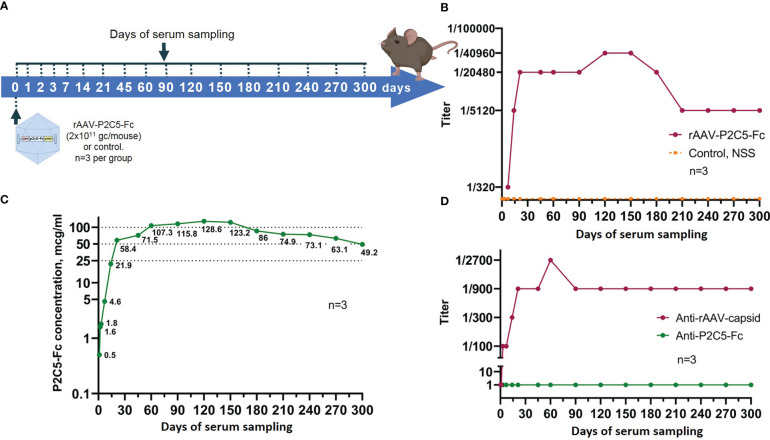
Evaluation of blood serum samples collected from mice treated with rAAV-P2C5-Fc. **(A)** Scheme of the experiment: a single intramuscular injection of rAAV-P2C5-Fc at a dose of 2*10^11^ gc/mouse was performed. Serum samples were taken at days 0, 1, 2, 3, 7, 14, 21, 45, 60, 90, 120, 150, 180, 210, 240, 270, and 300 after the injection; **(B)** Titers of virus-neutralizing antibodies to SARS-CoV-2 B.1.1.1 variant, **(C)** Levels of P2C5-Fс antibody and **(D)** Titers of rAAV- and P2C5-Fc-specific antibodies in blood serum of the animals. Lines – mean values, n – number of animals per group.

Scheme of the experiments as well as experimental results are presented in **Figures 8A, B**. A significant virus neutralization activity was detected already at the day 7 after the transduction (antibody titer of 1/320) and further increased to values of 1/5120, 1/20480 and 1/40960 on days 14, 21 and 120, respectively. It was also noted that high titer of virus neutralizing antibodies (1/40960) remained until day 150 after the transduction. Starting from the day 180 of the experiment level of the virus neutralizing antibodies decreased to 1/20480, reached value of 1/5120 on day 210 and remained unchanged within 300 days.

Generated data therefore indicate rapid achievement of extended virus neutralization activity of blood serum after a single rAAV-P2C5-Fc administration.

### 3.8 Evaluation of pharmacokinetics of P2C5-Fc antibody expressed by rAAV-P2C5-Fc

To evaluate pharmacokinetic properties of P2C5-Fc antibody female BALB/c mice were transduced with an rAAV-P2C5-Fc dose of 2*10^11^ gc/animal. In total 10 animals were used. Blood serum was collected at 0, 1, 2, 3, 7, 14, 21, 45, 60, 90, 120, 150, 180, 210, 240, 270 and 300 days after the transduction. Sampling scheme is summarized in **Figure 8A**.

As a result, P2C5-Fc was detected in serum already 48 hours following mice transduction with rAAV-P2C5-Fc and its level further significantly increased during the next 5 days. At the day 28 the antibody concentrations exceed 100 µg/mL and remained above this level until day 150. On days 180, 210, 240, 270 and 300 after transduction, the serum P2C5-Fc concentrations were 86, 75, 73, 63 and 49 µg/mL, respectively. Experimental results are shown in **Figure 8C**.

Summarizing all mentioned above it can be said that usage of rAAV-P2C5-Fc enables rapid accumulation and prolonged circulation of P2C5-Fc antibody within the living organism.

### 3.9 Evaluation of rAAV-P2C5-Fc immunogenicity

To evaluate immunogenicity of rAAV-P2C5-Fc blood serum samples from **Figure 8A** were used. Concentration of anti-idiotypic antibodies to P2C5-Fc antibody was below quantification limit whereas a significant level of antibodies specific to rAAV capsid proteins was detected on day 14, reached maximum on day 90 and remained detectable within at least 300 days after the transduction (**Figure 8D**).

Summarizing these observations, it can be said that no significant production of P2C5-Fc specific antibodies was observed but substantial level of the antibodies specific to rAAV capsid proteins was detected.

## 4 Discussion

Development of COVID-19 prophylaxis and treatment strategies remains relevant. Antigenic evolution and emergence of new variants of the virus with selective advantage and increased transmissivity driven by high antigen variability combined with natural selection leads to their rapid spread. This antigenic shift can lead to a decrease in efficacy of recently approved vaccines and therapeutic agents which emphasizes the relevance of new agents for prophylaxis and therapy of diseases caused by SARS-CoV-2 virus.

In a previous study we had used a bactrial camel immunization followed by selection of monoclonal antibodies with the highest phagocytic activity to generate a panel of specific monoclonal antibodies with the highest neutralizing capacity against concerning variants of SARS-CoV-2 virus (17). As a result, P2C5 antibody had been chosen for further studies based on virus-neutralizing concentration, equilibrium dissociation constants and specific activity to RBD of S glycoprotein, measured by immunofluorescence assay.

In current work we proposed an alternative immunization strategy implying the use of rAAV expressing a P2C5-Fc recombinant antibody to induce rapid long-term protection against SARS-CoV-2 viruses. A P2C5-Fc recombinant antibody represents a P2C5 antibody fused with an Fc-fragment of human IgG1 enabling its dimerization. This modification can increase avidity and specific activity as well as circulation time of the molecule due to an increase in molecular weight and interaction with neonatal Fc receptor for IgG (FcRn) (21). The proposed recombinant Fc-fusion single-domain antibody variant may be more effective than a monoclonal antibody due to the presence of long CDR3 loops enabling their binding to conservative hard-to-reach epitopes of viral antigens. Thus, such recombinant antibodies can have a wide range of neutralizing activity against various variants of the influenza virus, Ebola virus and SARS-CoV-2 (16, 17, 21). Thus, the emergence of antibody-resistant virus strains is less likely.

Application of rAAV as a vector for delivery, expression of genes encoding neutralizing antibodies and induction of passive immunity has been tested in prophylaxis of various infection diseases (11–15, 22). Selection of rAAV for delivery and expression of antibodies is dictated by their safety, low immunogenicity, ability to transduce a wide spectrum of different cells, prolonged persistence in a body in a form of episomes and inability of their integration into the host genome (23). A similar study demonstrating a prolonged expression of a recombinant antibody specific to botulotoxin type A following rAAV administration had been conducted previously by our group (15). This study also implied use of an antibody modified by an Fc fragment of human IgG1. We had demonstrated that the generated recombinant vector enables expression of the antibody in transduced mice for 4 months and therefore protect the animals from lethal doses of botulotoxin type A.

As in the previous aforementioned study in current work we used an rAAV-DJ system enabling derivation of a recombinant AAV with a hybrid serotype, which was produced and purified as described previously (15, 18). By analogy with the previous work an *in vitro* transduced activity of rAAV-P2C5-Fc and specific activity of P2C5-Fc expressed by rAAV were studied.

On the next step an effective dose of 2*10^11^ gc/mouse (10^13^ gc/kg) of rAAV-P2C5-Fc was selected based on the data from humanized mouse ACE-2 model of lethal infection. This finding is in line with previous studies where protective doses of rAAV preparations expressing antibodies ranged from 10^10^ to 10^12^ gc/animal (24–27). In a previous study we had investigated protective activity of a similar agent rAAV-B11-Fc which was 10^11^ gc/mouse. Lower protective dose of rAAV-B11-Fc vs rAAV-P2C5-Fc was attributable to lower neutralizing B11-Fc concentration against botulotoxin A vs neutralizing P2C5-Fc concentration against SARS-CoV-2 virus.

We further investigated protective capacity of rAAV-P2C5-Fc as an early and emergency pre-exposure as well as post-exposure prophylaxis. A humanized ACE-2 mice were transduced simultaneously (emergency prophylaxis) or 1, 3, 7 and 14 days before (early prophylaxis) or 16 hours after (post-exposure prophylaxis) SARS-CoV-2 В.1.1.1 variant injection for this purpose. Early and emergency COVID-19 prophylaxis with rAAV-P2C5-Fc was associated with full animal protection and lack of any disease symptoms in mice. At the same time a 40% survival rate and a significant body weight decrease was detected in an animal group received post-exposure prophylaxis. Development of the infection process followed by animal death takes about 7-8 days after the virus exposure and a significant P2C5-Fc accumulation in blood serum happens by that time in case of simultaneous administration of rAAV-P2C5-Fc and the virus. An accumulated P2C5-Fc enables virus neutralization and stopping the pathological process which can explain high efficacy of the rAAV-P2C5-Fc for emergency pre-exposure prophylaxis. This hypothesis can be confirmed indirectly by evaluation of P2C5-Fc pharmacokinetics in rAAV-P2C5-Fc-transdused mice. It was demonstrated that the levels of virus neutralizing antibodies in blood serum of mice on day 7 after rAAV-P2C5-Fc-transduction significantly exceeds those in blood of people recovered from a single COVID-19 episode (1/320 *vs* an about 1/200 neutralizing antibodies titer in convalescent plasma) (7). In addition, it was experimentally confirmed that protection against SARS-CoV-2 infection following mice transduction with rAAV-P2C5-Fc is mediated *via* the expression of a specific P2C5-Fc antibody, since mice transduced with rAAV-B11-Fc expressing B11-Fc antibody specific to botulinum toxin died as well as control mice after SARS-CoV-2 infection.

Evaluation of duration of rAAV-P2C5-Fc protective activity against SARS-CoV-2 B.1.1.1 variant was performed using the humanized ACE-2 mouse model. The study results were also in line with measured P2C5-Fc pharmacokinetic profile and a virus neutralizing activity of mouse blood serum collected from rAAV-P2C5-Fc transduced animals. The data generated in our study demonstrated that protection of the rAAV-P2C5-Fc-transduced mice against SARS-CoV-2 variant B.1.1.1 lasts at least 140 days following rAAV administration. Concentrations of both P2C5-Fc antibody and virus neutralizing antibodies in the blood serum were shown to rise sharply starting from day 7, reached plateau approximately on day 28 and remained stable till day 150 after the transduction. It is also important to note that the level of virus neutralizing antibodies to SARS-CoV-2 B.1.1.1 variant in mouse blood serum, measured starting from 28 days post transduction exceeded that from the blood serum of the COVID-19 convalescents and persons vaccinated with Sputnik V (6, 7). The possible influence of rAAV on the induction of non-specific protection in the long-term prophylaxis regimen cannot be completely ruled out. However, we believe this is unlikely, since Liao G. et al. (28) showed that the administration of a control rAAV expressing EGFP does not induce immunogenicity and protection against SARS-CoV-2 virus in a long-term prophylaxis regimen.

According to our study minimal neutralizing concentration of P2C5-Fc antibody against SARS-CoV-2 virus B.1.1.1 *in vitro* is about 6 ng/ml. Considering maximal neutralizing dilutions of the serum derived from rAAV-P2C5-Fc treated mice and *in vivo* concentrations of P2C5-Fc antibody the minimum neutralizing activity of P2C5-Fc antibody in the serum of rAAV-P2C5-Fc treated mice should be about 3-12 ng/ml, which is in line with *in vitro* data. In a previous study, we had shown that protective rAAV activity directly correlates with level of neutralizing antibodies in blood serum (15). Accumulated evidence therefore suggests that rAAV-P2C5-Fc provides protection against SARS-CoV-2 induced infection during the P2C5-Fc circulation, that is, at least 300 days, and, most likely, even more.

In our study the maximum concentration of P2C5-Fc antibody in the mouse blood serum on days 60-150 after administration of 2*10^11^ gc of rAAV-P2C5-Fc was about 100-130 μg/ml. In the work of Del Rosario et al. (22), several rAAVs expressing recombinant molecules consisting of single-domain antibodies fused with Fc-fragments of various mouse IgG isotypes were obtained. The antibodies demonstrated specific activity against various variants of influenza A virus *in vitro* and were used for *in vivo* immunization with rAAV at the doses of 10^10^, 3.3*10^10^ and 10^11^ gc per animal. As a result, Del Rosario and colleagues showed significantly higher expression of recombinant antibodies (500-1000 μg/ml) compared our results. This is most likely related to the fact that the authors used mouse IgG Fc-fragments, and the efficiency of their expression in mice can be greater compared to the human ones used in our study. In addition higher affinity of mouse IgG2A for murine Fc-receptors (29) can explain a sharper rise and a stronger accumulation of antibodies in the organism of mice observed in the Del Rosario et al. study. The authors also demonstrated that antibodies expressed after rAAV transduction circulate in the blood serum of mice for six months without a significant decrease. In our study, the antibody concentration in the blood serum of transduced mice began to noticeably decline only after day 150-180, which does not contradict the data of Del Rosario and colleagues.

To evaluate rAAV biodistribution following intramuscular drug administration we generated an rAAV expressing a luciferase gene (rAAV-Luc). Specific luciferase activity was detected solely at the site of the drug administration (the thigh muscles of the hind limb) which indicates local transduction of the muscle cells and lack of the systemic transduction under the treatment. Luciferase activity was detected for at least 60 days after the transduction, which indicates persistence of the rAAV expressing the target transgene for a long time. Prolonged persistence of rAAV in muscle cells as an episome for many months after a single intramuscular injection was shown in a non-human primate model by Penaud-Budloo M. et al. (30).

Investigation of rAAV-P2C5-Fc immunogenicity showed presence of antibodies to rAAV capsid proteins in a blood serum of transduced mice however no antibodies to P2C5-Fc were detected, which can explain much longer circulation of P2C5-Fc expressed by rAAV in the current study compared to rAAV-B11-Fc, expressing B11-Fc antibody, used in a previous study (15).

In our previous study we had also demonstrated protective capacity of P2C5 antibody against various SARS-CoV-2 variants including Omicron B.1.1.529. In current work we showed efficacy of SARS-CoV-2 Omicron BA.5 variant prophylaxis with rAAV-P2C5-Fc. As P2C5-Fc activity against Omicron BA.5 is lower compared to that against B.1.1.1, virus inoculation was performed at the day 20 after transduction to ensure sufficient antibody level at the time of exposure to the virus. We had demonstrated that minimal neutralizing concentration of P2C5-Fc antibody against SARS-CoV-2 B.1.1.1 and SARS-CoV-2 BA.5 was about 6 ng/ml and 1.2 μg/ml, respectively.

In conclusion it can be said that the efficacy of passive immunization with a recombinant adeno-associated viral vector rAAV-P2C5-Fc expressing a recombinant neutralizing antibody P2C5-Fc for protection against SARS-CoV-2 infection was first shown in our study. In addition, we demonstrated high protective capacity of rAAV-P2C5-Fc against various SARS-CoV-2 variants, including Omicron BA5. And finally, we showed 100% efficacy of the developed rAAV for an emergency prevention and long-term prophylaxis, respectively.

## Data availability statement

The original contributions presented in the study are included in the article/supplementary material. Further inquiries can be directed to the corresponding author.

## Ethics statement

The study was conducted according to the guidelines of the Guide for the Care and Use of Laboratory Animals published by the National Institutes of Health (NIH Publication #85–23, revised 1996) and National Standard of the Russian Federation GOST R 53434–2009, approved by the Institutional Animal Care and Use Committee (IACUC) of the Federal Research Centre of Epidemiology and Microbiology named after Honorary Academician N.F. Gamaleya and were performed under Protocol №25 dated Apr 22, 2022.

## Author contributions

Conceptualization: IE, DS, DL, BN. Methodology: IE, ER, AD, ID, IF, DG, AGo. Validation: IE, ER, AD, ID, DG. Formal analysis: ER, IE, AD. Investigation: ER, AD, IE, VP, MD, ID, DG, IZ, AI, AK, AS, AGo, D.V.B., IF. Resources, DL, AG, DS, BN, Data curation: IE. Writing – original draft preparation: IE, AD, ER. Writing – review & editing: IE, DS, ID. Visualization: AD, IE, ER, ID, DG. Supervision: IE. Project administration: DL, DS, BN, AGi. All authors contributed to the article and approved the submitted version.
